# Contraception and abortion information and care in community pharmacy for adolescents: a systematic review

**DOI:** 10.1016/j.eclinm.2025.103394

**Published:** 2025-07-31

**Authors:** Anisa Rojanapenkul Assifi, Danielle Mazza

**Affiliations:** Department of General Practice, SPHERE Centre of Research Excellence, School of Public Health and Preventive Medicine, Monash University, 553 St Kilda Road, Melbourne, VIC 3004, Australia

**Keywords:** Adolescent, Contraception, Abortion, Community pharmacy, Primary care, Sexual and reproductive health

## Abstract

**Background:**

Adolescents encounter greater barriers when accessing sexual and reproductive health (SRH) services than adults. Community-based pharmacists are uniquely positioned to address this due to their accessibility and availability within urban and rural contexts. This systematic review aimed to examine adolescents’ experience and acceptability of contraception and abortion clinical and dispensing services in community pharmacy.

**Methods:**

Seven databases were systematically searched to identify original peer-reviewed studies from high-income countries, from 2000 to 2025, relating to adolescents, pharmacy, contraception and medication abortion. The search was conducted in June 2023 and updated in April 2025. This review is registered with Prospero (CRD42022298209).

**Findings:**

Thirty-four studies were included; the majority focused on the emergency contraceptive pill. Despite adolescents feeling that the pharmacy was an accessible and convenient source of SRH services and that pharmacists provided them with comprehensive information, adolescents experienced and feared embarrassment, judgement and stigma.

**Interpretation:**

Though this review identified community pharmacies as safe, accessible settings where adolescents can conveniently seek care, competency in adolescent-friendly care needs to be optimised, with greater focus on expanding pharmacists’ scope-of-practice.

**Funding:**

No funding was received for this study.

## Introduction

Adolescence, defined by the World Health Organisation as 10–19 years, is a developmentally sensitive period of rapid social and cognitive development.^1^^,^^2^ Although the rate of pregnancy and birth amongst adolescents is declining, they are at higher risk of experiencing an unintended pregnancy. Accessibility of quality, adolescent-friendly sexual and reproductive health (SRH) services is key to ensuring positive health and socioeconomic outcomes for this group.^3^ Yet, despite adolescents comprising one-sixth of the world’s population,^1^ health systems cater poorly to their needs. Adolescents encounter greater barriers when accessing services than adults, including stigma, out-of-pocket costs, restrictive legislative frameworks, and judgement from community and healthcare providers.^4^^,^^5^ Many adolescents also lack SRH knowledge and have poorer health literacy than adults, with these two factors combining to delay their access to services.^6^^,^^7^ They may then encounter providers who hold stigmatised views and/or lack understanding of adolescent SRH needs at the point of care.^8^ These factors contribute to inequitable access to contraception through non-use or inconsistent use of contraception when it is wanted, leading to an unexpected pregnancy.^9^

To appropriately respond to the needs of adolescents and address the challenges that they face, it is important to make it easier and equitable for adolescents to access ‘friendly’ health services. Adolescent-friendly services (also referred to as ‘youth-friendly services’), are defined by the World Health Organisation as those that are accessible, acceptable, equitable, effective, and appropriate for adolescents.^10^ Ambresin et al. (2013) identified eight domains of adolescent-friendly healthcare important to adolescents: accessibility of healthcare; staff attitude; communication; medical competency (technical skills); guideline-driven care; age-appropriate environment; involvement in healthcare; and health outcomes.^11^

Efforts to bring about equitable and convenient access to SRH care for all people are leveraging the role and accessibility of community pharmacy services. While adolescents are a heterogeneous group, they consistently identify common characteristics essential to adolescent-friendly services across various settings—being treated with respect by healthcare providers and ensuring their confidentiality is maintained.^11^^,^^12^ For adolescents in particular, pharmacies offer a unique opportunity to be an entry point into the health system as they are widely available within both urban and rural communities and frequently accessed.^13^ In these interactions, pharmacists can provide adolescents with information and support before they secure an appointment in the primary care system or dispense contraception without the need for a prescription where this has been legislated.^14^ Regulations on pharmacy provision of contraceptives have eased in high-income countries^15^^,^^16^—in the United States (US), United Kingdom (UK) and Australia where emergency contraceptive pills (ECPs) are now available over the counter without age or gender barriers,^15^^,^^16^ and the US and UK where over-the-counter provision of hormonal contraceptives has been introduced.^17^ These recent changes reduce barriers that exist for adolescents seeking contraception, although out-of-pocket costs for medications continue to be a challenge.^18^^,^^19^

While small initiatives are being undertaken across various countries and settings (including in community pharmacy) to overcome barriers and deliver quality ‘friendly’ health services to adolescents, there is still a limited understanding around the delivery of SRH services to adolescents through the pharmacy setting.^12^ While a previous systematic review focused on young people (≤25 years) and their experiences with SRH information and services in the pharmacy setting,^20^ none have focused specifically on the adolescent population. To better understand the status of adolescent-friendly services in community pharmacy and how to improve care, this systematic review aimed to examine adolescents’ experience and acceptability of contraception and abortion clinical and dispensing services in community pharmacy.

## Methods

Methods are reported according to the PRISMA 2020 statement.^21^ This review is registered with Prospero (CRD42022298209).^22^

### Outcomes

The review focused on two outcomes: acceptability and experience. The term ‘acceptability’ is defined as a “multi-faceted construct that reflects the extent to which people delivering or receiving a healthcare intervention consider it to be appropriate based on anticipated or experienced cognitive and emotional responses to the intervention”.^23^ We were also interested in the experience of accessing contraception and abortion services in community pharmacies from adolescents’ perspectives and the provision of these services to adolescent populations from pharmacist/pharmacy staff’s perspectives. Experience relates to the actual interaction between adolescents and pharmacists or pharmacy staff, as well as the community pharmacy setting.

### Inclusion criteria

The full inclusion and exclusion criteria are provided in Table 1. Eligible studies were those published from the year 2000 onwards due to the significant shift in pharmacists’ scope of practice since 2000 in high-income countries. Additionally, studies of any design were included provided they focused on or included disaggregated data on adolescents aged 10–19 years. Studies examining medication dispensing rates and sexually transmissible infections (STIs) were excluded.

**Table 1:** Inclusion criteria.

Inclusion	Exclusion
Original/primary research study with either qualitative or quantitative data, or both	Discursive/descriptive, incidence, prevalence, grey literature
Studies including adolescents 10–19 years or focused provision of services to adolescents	
Experience or acceptability of receiving/providing care to adolescents in the pharmacy setting	
Dispensing of and provision of information on contraception and abortifacients (medication abortion medicines)	STI testing kits Prevalence of pharmacy provision
English	
Studies published since 2000	
High-income countries^24^	

### Search strategy

A systematic search of original research published in peer-reviewed journals was conducted in seven databases (CINAHL, Embase, Medline, PsychInfo, Scopus, Pubmed, Web of Science) and the Cochrane database in June 2023, updated April 2025. Reference lists of included studies were also manually searched. Following consultation with a medical librarian and piloting of search terms, controlled vocabulary keywords (MeSH) and free-text terms including–‘adolescent’, ‘pharmacy’, ‘abortion’, and ‘contraception’, along with their synonyms and relevant medications, and device name–were used to search the databases. Two test studies were used to determine the comprehensiveness of the search strategy.^25^^,^^26^

### Selection and screening process

Two researchers (AA and KH) independently screened titles and abstracts for inclusion, then reviewed the full-text studies using the inclusion criteria. Differences were discussed between the two researchers, and agreement was reached.

### Data extraction and synthesis

Data were extracted according to 1) publication characteristics, 2) study design, setting and methodology, 3) characteristics of the study population (e.g., age, sociodemographic data), and 4) study findings relevant to the review’s aims and outcomes. This was conducted by AA, then checked and verified by AS. Included studies were a range of qualitative and quantitative study designs across heterogeneous contexts, and therefore, a meta-analysis was not achievable. We undertook a narrative synthesis to analyse extracted data from the included studies. A convergent integrated approach was taken, where quantitative data were translated into textual descriptions to allow integration with qualitative data. Findings from each study that were relevant to this review were coded, resulting in the development of a set of codes. These codes were then organised into categories and mapped to the review outcomes of ‘acceptability’ and ‘experience’.

### Critical appraisal

A study-level assessment of the methodological quality of the included studies was undertaken using the JBI Critical Appraisal tools.^27^ Critical appraisal was undertaken by one researcher and independently verified by a second researcher. Differences were discussed between the two researchers, and an agreement was reached (Supplementary Table S1). Study design-specific checklist tools were used to identify methodological issues, with methodological quality based on the percentage of “yes”, “no” and “unclear” responses to each question. A “yes” response indicated adequate reporting on the checklist item. Studies with 0%–49% “yes” responses were deemed low-quality, 50%–69% moderate quality, and 70%–100% high-quality.^28^, ^29^, ^30^

### Role of the funding source

No funding was received for this study.

## Results

**Fig. 1:**
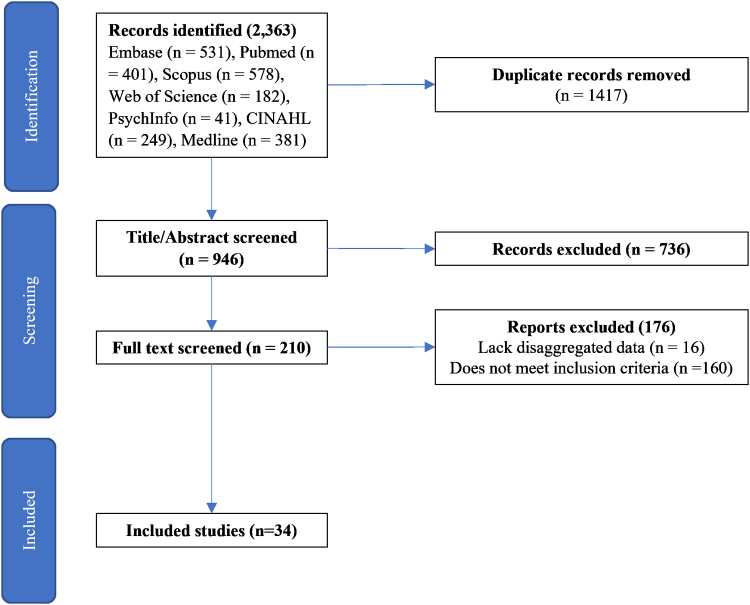
Flow diagram of study selection process.

The search yielded 2363 citations. After excluding duplicates, 946 studies were screened for inclusion based on title and abstract. Of these, 210 studies were read in full to assess if they met the inclusion criteria. Thirty-four studies were included in the analysis (Fig. 1).

Of the 34 included studies (Table 2), the majority were from the US (n = 24), four from the UK, two from Australia, and one each from Switzerland, Canada, and New Zealand. The emergency contraceptive pill (ECP) was the focus of 21 of the 34 studies, and 10 studies focused broadly on contraceptive methods. Provision of contraception and youth-friendly services at the pharmacy was the aim of 3 studies. The majority of included studies (n = 27) described patients’ perspectives or experiences (real or simulated), with five of these studies solely focused on adolescents. There were 24 quantitative studies, eight qualitative studies and two mixed-methods studies. Thirteen studies were published in the last five years, and nine were published before 2013. None of the studies pertained to abortion medication dispensing. Table 3 presents findings synthesised by adolescents and pharmacist/pharmacy staff acceptability and experience.

**Table 2:** Characteristics of included studies (N = 34).

Author/Year	Country	Study period	Study design and methods	Study population	Setting	Aim	National regulations at the time of the study
Wilson & Williams 2000^31^	United Kingdom	1995–1997	Quantitative (school- and mail-based survey)	Adolescent Females and males aged between 13 and 19 years (n = 711)	Administered in two of the three local comprehensive Schools to 13–16-year-olds and sent through the post using the age–sex registers of five of the seven practices in the locality in Leicester, UK, for 16–19-year-olds to complete.	To examine the current provision of contraception to teenagers in the locality and their views about how this could be improved, particularly to assess the need for a young people’s service based in a local community centre.	The oral contraceptive pill is available only with a prescription.
Sucato et al. 2001^32^	United States	June and October 1999	Quantitative (self-administered survey)	Adolescent included 15–21 years old (n = 126) who obtained ECP directly from a pharmacist.	Fifteen randomly selected pharmacies providing ECP in western Washington State.	To increase knowledge about adolescents who obtained ECP directly from a pharmacist without first contacting a physician.	ECP is only available through prescription by a doctor. Pharmacists included were part of a state-based program and were being trained in ECP prescribing in the hope of making ECP available to women without a prescription.
Conard et al. 2003^25^	United States	Not stated	Quantitative (self-administered, mail-in survey)	Pharmacist Chief pharmacists (n = 948)	All active licensed pharmacies in Indiana, US.	To describe pharmacists’ attitudes, dispensing practices, and perceived adequacy of training related to adolescent patients. Specific attention was given to the provision of reproductive health services such as contraceptives.	ECP is only available through prescription by a doctor.
Lewington & Marshall 2006^33^	United Kingdom	December 2002–October 2003	Qualitative (pharmacy record review)	Adolescent Women <20 years of age (n = 203) requesting ECP from family planning clinics and community pharmacies	Community pharmacies in South-West Kent Primary Care Trust	To evaluate differences in the time taken to access progestogen-only emergency hormonal contraception (EHC) by young women from family planning (FP) or community pharmacy settings.	In the study area, ECP can be obtained for free if they are aged less than 20. Levonorgestrel-only ECP could be obtained over the counter (OTC) by women over the age of 16.
Hobbs, Taft & Amir 2009^34^	Australia	February–June 2007	Qualitative (focus groups)	Adolescent included Women aged 16–30 years agreed to participate in the focus group (n = 29). Participants aged between 16 and 20 years (n = 13)	Adolescents living in four Australian states/territories (Victoria, New South Wales, Northern Territory and Queensland)	To explore Australian women’s knowledge, attitudes and experience of the ECP since becoming available over the counter.	ECP is available OTC. In Australia, pharmacists can choose to provide adolescents under the age of 16 with the ECP if they believe it is appropriate.
Sampson et al. 2009^35^	United States	August 2005–April 2006	Mixed-method (quantitative mystery-client and qualitative interviews) Three female researchers posed as English or Spanish-speaking 15-year-olds who had unprotected sex the previous night or 18-year-olds who had unprotected sex 4 days earlier.	Pharmacist & Clinician Pharmacies in California (n = 115) Interviews were carried out with clinicians (n = 13) and pharmacists (n = 9) on their experiences.	Community pharmacies in California, US	To consider the role of pharmacy access to emergency contraception in reducing unintended pregnancy among adolescents, and focus on language barriers to such access.	Since 2002 in California, adolescents <18 have been able to access levonorgestrel- ECP from designated pharmacies without a clinician’s prescription. In August 2006, the U.S. Food and Drug Administration (FDA) permitted pharmacists to dispense ECP without a physician’s prescription to women ≥18 (with proof of age).
Rubin et al. 2011^36^	United States	February–April 2009	Quantitative (self-administered web-based survey)	Adolescent Females aged between 14 and 19, living in the US and had ever engaged in unprotected intercourse at a time when they were aware of ECP (n = 531)	Web-based national survey	To examine the effect of policies regarding access to emergency contraception on teens’ promptness of ECP use and satisfaction with ECP access and experience	ECP is accessible without age limits in 9 states. In all other states, women needed to be either over 17 or 18. In 2009, the US Food and Drug Administration made levonorgestrel-ECP available without a prescription to individuals ≥17 years; those <17 years required a prescription
Richman et al. 2012^37^	United States	February–June 2008	Quantitative (self-administered, mail-in survey)	Pharmacist Pharmacists practicing in Florida, US (n = 272)	Randomly selected pharmacists practicing in Florida and registered with the Florida Board of Pharmacy.	To investigate the extent to which knowledge and attitudes predict ECP dispensing among a state sample of Florida pharmacists.	ECP can be purchased over-the-counter for people over the age of 18. State law allows pharmacists to refuse dispensation.
Wilkinson et al. 2012^38^	United States	Not stated	Quantitative (mystery client) Mystery caller posed as a 17-year-old or a physician calling on behalf of a 17-year-old.	Pharmacy staff Pharmacies (n = 943) in five cities in the US.	Every community pharmacy in Nashville, Tennessee; Philadelphia, Pennsylvania; Cleveland, Ohio; Austin, Texas; and Portland, Oregon.	To assess the accuracy of information provided to adolescents and their physicians when they telephone pharmacies to inquire about ECP.	In 2009, the US Food and Drug Administration made levonorgestrel-ECP available without a prescription to individuals ≥17 years; those <17 years require a prescription.
Parsons et al. 2013^39^	United Kingdom	April–May 2010 (mystery client component)	Quantitative (mystery client, medical records and self-administered survey)	Pharmacy staff Pharmacies (n = 3) in Lambeth and Southwark, UK	Three community pharmacies in Lambeth and Southwark that had submitted an expression of interest in providing oral contraceptives.	To evaluate the oral contraception service delivered by community pharmacists in Lambeth and Southwark.	In 2009, Southwark and Lambeth Primary Care Trusts (PCTs) developed a patient group direction (PGD) for community pharmacists to supply combined oral contraceptives and progestogen-only pills to women >16 years who fulfilled particular criteria, without a prescription.
Horsfield et al. 2014^40^	New Zealand	May–September 2011	Quantitative (self-administered, mail-in survey)	Pharmacy staff Pharmacist (n = 251) and pharmacy support staff (n = 184)	Community pharmacies were randomly selected from the New Zealand Pharmacy Guild’s national database of community pharmacies.	To investigate the availability of youth-relevant community pharmacy services in New Zealand and the opinions of pharmacy personnel on the appropriateness of these services for young people aged 12–24.	There is no age restriction on the provision of ECP from pharmacies.
Wilkinson et al. 2014^41^	United States	September–December 2010	Qualitative (mystery client) Mystery caller posed as a 17-year-old or a physician calling on behalf of a 17-year-old.	Pharmacy staff Commercial pharmacies in five United States cities (n = 943)	Every community pharmacy in Nashville, Tennessee; Philadelphia, Pennsylvania; Cleveland, Ohio; Austin, Texas; and Portland, Oregon.	To understand the experiences of adolescent females when they try to obtain emergency contraception from pharmacies.	In 2009, the US Food and Drug Administration made levonorgestrel-ECP available without a prescription to individuals ≥17 years; those <17 years require a prescription.
Hussainy, Steward & Pham 2015^42^	Australia	January 2013	Quantitative (mystery client) A mystery caller posed as someone who had had unprotected intercourse greater than 72 h ago (Scenario 1), a 16-year-old (Scenario 2) and someone who was requesting ECP for future use (Scenario 3).	Pharmacy staff Pharmacies allocated to scenario 1 (n = 166), pharmacies to scenario 2 (n = 167) and pharmacies to scenario 3 (n = 167).	Community pharmacy in Victoria, Australia	To determine emergency contraception supply practices of a sample of pharmacies in Victoria, Australia, using mystery client evaluation methodology	There is no reason to restrict the provision of ECP based on age if the pharmacist believes the person is mature and that their health would suffer without the treatment or advice.
Manski & Kottke 2015^9^	United States	September 2014	Quantitative (self-administered, web-based survey)	Adolescent Female adolescents aged 14–17 (n = 348)	Web-based national survey	To assess female teenagers’ attitudes toward oral contraceptives being available for teenagers over the counter, as well as through pharmacy access (another provision model that may expand use) and their understanding of a prototype over-the-counter product label.	At the time of the study, a prescription from a doctor was required to obtain hormonal contraceptives.
Cleland et al. 2016^43^	United States	2015	Quantitative (self-administered, web-based survey)	Pharmacy Pharmacies across 23 states in the US (n = 220)	Pharmacies and stores with pharmacy sections across the US	To describe the state of access to fully over-the-counter levonorgestrel emergency contraception.	Levonorgestrel ECP available over-the-counter in pharmacies in the US with no gender or age restrictions since June 2013. Ulipristal acetate ECP is a prescription-only product.
Wilkinson et al. 2017^44^	United States	July–December 2015	Quantitative (mystery client) Mystery callers posed as 17-year-olds seeking ECP.	Pharmacy staff Pharmacies in five United States cities (n = 979).	Every community pharmacy in Nashville, Tennessee; Philadelphia, Pennsylvania; Cleveland, Ohio; Austin, Texas; and Portland, Oregon.	To examine if the US Food and Drug Administration (FDA) policy change resulted in increased availability of or access to ECP for adolescents by using the same study design and population as the Wilkinson et al., 2012 study.	Levonorgestrel-ECP available over-the-counter in pharmacies in the US, with no gender or age restrictions, since June 2013.
Ritter et al. 2018^45^	United States	January–February 2015	Quantitative (mystery client) One male and one female volunteer posed as a 17-year-old to make the calls	Pharmacy staff Pharmacies were contacted and asked for information regarding ECP (n = 90).	Community pharmacies in Richmond, Virginia, that supply Plan B One Step to the public.	To discover if there are barriers to access and to determine if such barriers vary based on the gender of the person making the purchase.	Levonorgestrel ECP available over-the-counter in pharmacies in the US with no gender or age restrictions since June 2013.
Wilkinson et al. 2018^46^	United States	July–September 2015	Qualitative (interviews)	Adolescent English-speaking females aged between 18 and 19 in California (n = 30)	Adolescents living in California.	To describe adolescent attitudes toward access to contraception using the traditional prescription model, as well as their attitudes and interests in pharmacist prescribing of non-long-acting reversible contraception before it was implemented in California	Six states in the US permit pharmacists to prescribe contraceptives; California is the only state to allow adolescents under the age of 18 to use this program.
Wilkinson et al. 2018^47^	United States	July 2015–January 2016	Quantitative (mystery client) There were three sets of mystery callers comprising 2 female physicians, 2 adolescent females (17-year-olds) and 2 adolescent males (17-year-olds).	Pharmacy staff Pharmacies in five US cities (n = 993).	Licensed pharmacies in five different cities (Nashville, Tennessee; Philadelphia, Pennsylvania; Cleveland, Ohio; Austin, Texas; and Portland, Oregon).	To determine if access barriers to emergency contraception still existed and to evaluate variations in information offered by pharmacies utilizing three types of mystery callers: an adolescent female, an adolescent male and a female physician.	Levonorgestrel ECP available over-the-counter in pharmacies in the US with no gender or age restrictions since June 2013.
Uysal et al. 2019^48^	United States	Mid-April—mid-June 2016	Quantitative (mystery client) Male and female researchers phoned pharmacies posing as 16-year-olds requesting ECP.	Pharmacy staff Pharmacies were included in the final sample (n = 1475).	Community pharmacies in four southwestern states in the US—Arizona, California, New Mexico and Utah.	To evaluate the availability and accessibility of emergency contraception to adolescents in US pharmacies across four Southwestern states, 3 years after the federal Food and Drug Administration removed age restrictions for over-the-counter sales of levonorgestrel-only pill.	There is no age restriction for the over-the-counter sale of levonorgestrel-ECP.
Zuniga et al. 2019^49^	United States	February 2017	Qualitative (focus group)	Adolescent included Female 14–24 years, living, working or attending school in Washington, DC and interested in participating in a study about access to birth control (n = 31). 14–17-year-old participants (n = 14), 18–24-year-old participants (n = 17).	Females living in Washington, DC	To inform the implementation of pharmacist prescribing of contraception by exploring the needs and perspectives of teens and young adult women aged 14–24 years in Washington, D.C.	Pharmacists in certain areas of the US can now prescribe oral contraceptives OTC due to new legislation. This is not the case yet in the study location.
Ashcraft et al. 2020^50^	United States	September 2019–February 2020	Quantitative (mystery client) 14 female undergraduate and graduate students either posed as a transparent researcher or a 16-year-old mystery caller. Part of a larger study^51^^,^^52^	Pharmacy staff Community pharmacies throughout West Virginia (n = 509)	Community pharmacies in West Virginia, US	To assess the availability and accessibility of levonorgestrel emergency contraceptive, as well as the pharmacy staff’s knowledge regarding effectiveness and proper use of levonorgestrel emergency contraception.	ECP is available over-the-counter to anyone regardless of age.
Hsu et al. 2020^53^	United States	Mid-April—mid-June 2016	Quantitative (mystery client) 15 university students (10 female and 5 male) posed as 16-year-olds requesting information about female condoms at pharmacies.	Pharmacy staff Community pharmacies in four southwestern states in the US (n = 1475)	Community pharmacies in four southwestern states—Arizona, California, New Mexico and Utah.	To assess female condom availability by pharmacy type in southwestern states and describe heterosexual adolescent experiences when inquiring about female condoms, after first asking if there were any products to prevent pregnancy after unprotected sex.	Female condoms are the only FDA-approved alternative to male condoms.
Meredith et al. 2020^26^	United States	Not stated	Qualitative (interviews)	Adolescents included Female aged 14–21 (n = 60)	Adolescents in Indiana.	To identify how adolescents in Indiana perceive pharmacist prescribing of contraception	Pharmacies are now permitted to prescribe over-the-counter contraceptives to women in certain states of the US.
Soper et al. 2020^54^	Canada	June 2016–October 2016	Quantitative (self-administered, web-based survey)	Pharmacist Pharmacists who worked in community pharmacy within the last year (n = 591)	Community pharmacies across Quebec, Canada.	To evaluate the accessibility of levonorgestrel emergency contraception for adolescents in Quebec community pharmacies	In 2008, levonorgestrel-ECP was available over-the-counter in Canada except in the provinces of Quebec and Saskatchewan, where consultation with a pharmacist is required.
Stone et al. 2020^17^	United States	Not stated	Quantitative (self-administered, web-based survey)	Pharmacist Pharmacists in 21 states in the United States (n = 823)	Random sample of retail pharmacists in 21 states in the US (Arizona, California, Colorado, Florida, Georgia, Hawaii, Iowa, Illinois, Indiana, Massachusetts, Maryland, Maine, Michigan, Minnesota, North Dakota, New Hampshire, Oklahoma, Rhode Island, South Carolina, Tennessee and Wyoming).	To describe pharmacist perceptions of training and preparation to prescribe hormonal contraception, identify training gaps and elicit preferred training methods.	Trained pharmacists can now prescribe hormonal contraceptives in six states to anyone regardless of age.
Wollum et al. 2020^55^	United States	February 2017 and January–June 2018	Mixed method (focus group discussion and self-administered web-based survey)	Pharmacist Pharmacists participated in the focus group discussions (n = 6), and pharmacists participated in the online survey (n = 82).	Community and outpatient pharmacists in Washington, DC	To assess pharmacists’ interest, comfort level, training needs and barriers to prescribing hormonal contraceptives, particularly in the context of serving young people in Washington, DC	Hormonal contraceptives can be prescribed by pharmacists to people of all ages.
Glasier et al. 2021^16^	United Kingdom	April 2018–January 2019	Qualitative (mystery client) Mystery shoppers were female volunteers aged 16 years and older who received £20 for each completed visit.	Pharmacy staff Mystery shopper encounters across the 30 trial pharmacies in London (n = 32), Dundee and Edinburgh (n = 23) (total n = 55)	Pharmacies in Edinburgh, Dundee and London	To evaluate the quality-of-service provision in community pharmacies and to determine what advice was being given about contraception after emergency contraception use.	ECP is available at study pharmacies without a prescription and is available for anyone over the age of 13.
Khorsandi et al. 2021^15^	United States	July 2018–January 2019 (female caller) October 2018–November 2019 (male caller)	Quantitative (mystery client) Two female researchers and one male researcher posed as 17-year-olds or clinicians seeking ECP on behalf of adolescents.	Pharmacy staff Pharmacists or pharmacy staff in pharmacies in 5 Louisiana cities in the US (n = 182).	Pharmacies in 5 cities in Louisiana, US.	To examine pharmacy-related barriers to adolescents’ access to emergency contraception in Louisiana	Louisiana does not have laws that allow pharmacists to refuse dispensation of ECP, but it also does not mandate dispensation.
Ashcraft et al. 2022^51^	United States	September 2019–February 2020	Quantitative (mystery client) (1) self-identified research calling to ask about LNG EC, and (2) a mystery caller, a member of the research team, posing as a 16-year-old seeking ECP Part of a larger study^50^^,^^52^	Pharmacy staff Pharmacy staff in community pharmacies (n = 509)	Community pharmacies in West Virginia, US	To assess the availability and accessibility of levonorgestrel emergency contraception at community pharmacies in West Virginia.	
Barrense-Dias et al. 2022^56^	Switzerland	April–August 2019	Qualitative (interviews)	Adolescent included Females aged 15–25 (n = 30)	Females living in the Canton of Vaud, Switzerland	To explore the experiences of adolescent and young adult females who have gone to a pharmacy to obtain emergency contraception	Emergency contraception access was liberalised in 2002.
Gomez et al. 2022^57^	United States	November 2019–May 2020	Quantitative (self-administered, web-based survey)	Adolescent included 15–44-year-olds assigned female sex at birth, residing in Tulare County, California (n = 177) 15–17-year-olds (n = 31)	Females living in Tulare County, California.	To describe community members’ awareness of, attitudes toward, interest in, and comfort with pharmacist-prescribed contraception in a rural California community.	California passed legislation authorising pharmacist prescribing of hormonal oral contraception in 2013, with services commencing in 2016. Pharmacist must complete training before prescribing oral, transdermal, vaginal and injectable contraception.
Ashcraft et al. 2023^52^	United States	September 2019–February 2020	Quantitative (mystery client) Mystery caller, 14 undergraduate and graduate female students, posing as a 16-year-old seeking ECP Part of a larger study^50^^,^^51^	Pharmacy staff Community pharmacies throughout West Virginia (n = 506)	Community pharmacies in West Virginia, US	To examine the availability and accuracy of pharmacy staff responses to questions asked about levonorgestrel ECP by our “16-year-old” mystery callers.	
Grindlay et al. 2023^58^	United States	January 2020–September 2021	Quantitative (self-administered, web-based survey)	Adolescent included Individuals who completed the ACCESS study (n = 665) Adolescent participants (n = 115)	Follow-up survey with ACCESS study participants at trial completion.	To assess participants’ experiences using a progestin-only pill in an over-the-counter setting, including how they felt about the menstrual bleeding they experienced, how participants experience the progestin-only pill compared with prior contraceptive methods and their preferred way to get answers to questions during over-the-counter progestin-only pill use.	The over-the-counter progestin-only pill was not available in the US at the time of the study.

**Table 3:** Findings synthesised by acceptability and experience.

Acceptability	Experience
**Adolescents** Main concerns that adolescents had about accessing contraception from a pharmacist were the embarrassment^31^ Pharmacists were seen by adolescents to possess sufficient knowledge when it comes to contraception and ECP^26^ Some adolescents viewed that the discussion around contraceptive methods during the provision was not at a convenient time and that it should only be provided after the ECP was taken^56^ The pharmacy was adolescents’ preferred source of information for questions about the over-the-counter progestogen-only pill^58^ Adolescents were very satisfied with the pharmacy service. They felt that obtaining contraception from the pharmacy was acceptable as it would be more convenient and accessible, save time and not require appointments^9^^,^^26^^,^^32^^,^^33^^,^^36^^,^^39^^,^^46^^,^^49^^,^^57^ Pharmacist prescribing and provision of over-the-counter oral contraceptives was acceptable to the majority of adolescent participants in the included studies^9^^,^^26^^,^^57^ A concern was that there is potential for a lack of continuity of care and a lack of confidentiality. However, in other included studies, the majority of adolescents identified they were very satisfied with the amount of privacy in the pharmacy setting^26^^,^^31^^,^^46^^,^^49^^,^^56^ **Pharmacist/Pharmacy staff** The acceptability of contraception provision among pharmacists, including dispensing ECP, varied across and within studies.^35^^,^^37^^,^^38^^,^^40^, ^41^, ^42^^,^^51^^,^^54^ Acceptability of ECP declined and was viewed as less appropriate for younger adolescents (<16 years).^40^ Pharmacists did feel comfortable providing counselling to adolescents, however, pharmacists also identified the need for additional training around how to provide information and care to adolescents and young people specifically, alongside how to help them select the best method of contraception^25^^,^^55^ Pharmacists were interested in prescribing hormonal contraception to adolescents^55^ Pharmacists reported feeling less comfortable providing adolescents with ECP than adults, and more comfortable dispensing to older adolescents (e.g., 17-year-olds) than younger adolescents (e.g., 14-year-olds)^37^^,^^54^ Pharmacy staff’s personal beliefs made them unwilling and unhelpful to provide contraceptive care, information or access to adolescents. With pharmacy staff refusing to assist adolescents’ access^38^^,^^41^^,^^42^^,^^51^ In some studies, pharmacist participants did not feel that the pharmacy is an acceptable setting to provide counselling when it comes to ECP provision^35^	**Adolescents** Pharmacy staff were seen to support adolescents’ accessibility to emergency contraception by offering to order medication^47^^,^^51^ The type of pharmacy (e.g., large chain versus independently owned) did impact the accessibility of emergency contraception due to higher correct knowledge around the need for prescription, identification requirement and obtaining without parental knowledge^50^ In some studies, adolescents identified that empathy and use of appropriate language during conversation improved their comfort and experience of accessing care, it supported their discretion^26^^,^^49^ Empathy was seen to greatly impact on experience, as did the gender of the pharmacist^26^^,^^46^^,^^49^^,^^56^ Adolescents had a fear of feeling embarrassed when interacting with pharmacy staff and of potential parental notification^36^^,^^49^ In some studies, adolescents reported experiencing embarrassment due to privacy concerns. In other studies, the embarrassment was due to dismissive, unhelpful and/or negative behaviour and treatment by pharmacy staff. This created negative experiences for adolescents^34^^,^^35^^,^^53^^,^^56^ Adolescents experienced and perceived to experience judgment by pharmacy staff^49^^,^^56^ Adolescents were provided detailed information, and they viewed pharmacists as knowledgeable sources^32^^,^^39^^,^^46^^,^^49^^,^^52^^,^^56^ Adolescents found that the pharmacy setting provided them with greater privacy and discretion when accessing contraception^49^ Adolescents’ experiences differed, with some pharmacy staff guaranteeing their confidentiality and privacy, and others not^41^ The pharmacy layout was identified as not conducive to privacy, resulting in unpleasant experiences for adolescents^49^ ‘False’ barriers created by pharmacists create challenges, making it more difficult and stressful for adolescents trying to access contraception. These ‘false’ barriers included their age, gender, institutional requirements, parental consent and need for prescriptions^16^^,^^25^^,^^35^^,^^38^^,^^45^^,^^51^^,^^53^ The interaction and the way care was provided by pharmacists and pharmacy staff were not always homogenous, resulting in adolescent discomfort^48^^,^^56^ The environment was not always adolescent-centred, which also resulted in adolescent discomfort when accessing information or contraception^34^^,^^56^ **Pharmacist/Pharmacy staff** Pharmacists acknowledge that pharmacists should not be judgmental or assume they know adolescents’ needs when providing information^55^ To reduce embarrassment experienced by adolescents, pharmacy staff suggested a mechanism where they could schedule an appointment in the pharmacy^55^ Pharmacists identified that providing checklists as a way to convey confidential information or information that the adolescent might feel uncomfortable or embarrassed to say could improve adolescents’ experience^55^ Pharmacists believed that it was important to ensure that there is a mix of pharmacists of different genders working at the same time, as adolescent females may experience embarrassment approaching and talking to male pharmacists and pharmacy staff^55^ Pharmacy staff lacked knowledge of the local legal requirements in relation to ECP to adolescents, e.g., whether parental consent was required to provide ECP to adolescents under the age of 18 and whether prescription was a requirement^15^^,^^35^^,^^38^^,^^41^^,^^43^, ^44^, ^45^^,^^47^ Pharmacists felt uncomfortable providing contraceptives to adolescents, especially younger adolescents^25^

Thirty-two studies were determined to be high-quality and two studies moderate quality (Supplementary Table S1).^17^^,^^40^ Factors that were not or unclearly addressed were the identification of confounding factors and strategies to deal with them (cross-sectional studies) and the influence of the researcher (qualitative studies).

### Acceptability

Both adolescents and pharmacists believed that pharmacy delivery of contraceptive services for adolescents was acceptable, as it was an accessible service where pharmacists were viewed as knowledgeable sources of information.

Adolescent participants generally viewed pharmacists as knowledgeable and acceptable sources of information about contraception and they were identified as a preferred source of information compared to other health professionals.^26^^,^^39^^,^^58^ However, the provision of contraception information during ECP counselling was not viewed as an appropriate time by adolescents in a study in Switzerland due to their concerns around delays in taking the ECP.^56^

The accessibility and convenience of pharmacies made it an acceptable source of SRH services for adolescents, especially when it came to accessing ECP, contraceptive information and methods.^26^^,^^32^^,^^33^^,^^36^^,^^46^^,^^49^ Pharmacist prescribing of contraceptive methods was also seen as acceptable by adolescents^9^^,^^26^^,^^57^ as it was seen to be beneficial to adolescents and could potentially increase the use of oral contraceptives. However, adolescents anticipated that pharmacists and pharmacy staff would exhibit judgemental attitudes,^31^ and they were concerned about pharmacists maintaining their confidentiality and the poor privacy in pharmacies due to the typical layout where there is no private space to discuss sensitive issues.^26^^,^^31^^,^^46^

Lack of access to medical history by pharmacists was identified in two studies as a reason why some adolescents did not view community pharmacists as an acceptable source of contraceptive prescribing.^26^^,^^49^ Adolescents felt that without knowledge of their medical histories, pharmacists may not be able to make the most informed recommendations for potential contraceptive methods. Pharmacists’ lack of access to adolescents’ medical information was also viewed as preventing continuity of care between the pharmacist and the adolescents’ doctors. Similarly, pharmacists in a mixed-method study found it unacceptable to provide adolescents with ECP because of the time required to deliver ECP counselling and the lack of access to adolescents’ medical records, which impacts on continuity of care.^35^

The acceptability of contraception provision among pharmacists, including dispensing ECP, varied across and within studies.^35^^,^^37^^,^^38^^,^^40^, ^41^, ^42^^,^^51^^,^^54^ Pharmacists’ and pharmacy staff’s attitudes toward providing information and dispensing contraception to adolescents appeared to be influenced by their attitudes toward contraception, age and adolescent sexual activity,^38^^,^^41^^,^^42^ with pharmacists and pharmacy staff not finding it acceptable to dispense contraception and ECP to adolescents.^35^^,^^37^ Over 90% of pharmacists in New Zealand found dispensing ECP over-the-counter to adolescents (16–18 years old) acceptable, but less appropriate for younger adolescents (<16 years).^40^ In US mystery shopper studies, pharmacy staff more often refused to assist adolescents in accessing ECP compared to when a physician called seeking ECP for an adolescent patient.^38^^,^^51^ In contrast, pharmacists in a Canadian study were less comfortable prescribing ECP to adolescents than adults, however, they still prescribed it to adolescents 99% of the time.^54^

Pharmacists themselves identified the need for additional training when providing contraceptive information and interacting with adolescents. Despite the majority (93%) of pharmacists in one study stating that they were comfortable counselling adolescents on hormonal contraception, over half wanted additional training on how to prescribe and counsel adolescents in particular.^55^ This was similarly observed in a separate study where only 13% of pharmacists felt well trained in adolescent-specific issues.^25^

### Experience

Adolescents experienced embarrassment and perceptions of pharmacist judgement, which negatively impacted their overall experience of accessing SRH services in pharmacies and were deterrents to future engagement in these services. They described concern about feeling embarrassed due to the poor privacy in pharmacies,^34^^,^^56^ awkwardness when interacting with the pharmacist/pharmacy staff,^34^^,^^36^ and judgement from the pharmacist/pharmacy staff.^38^^,^^41^^,^^42^^,^^49^^,^^51^^,^^56^ Feelings of embarrassment and judgement were made worse by pharmacy staff’s lack of sensitivity, which participants characterised as staff not being discreet (e.g., speaking loudly) during their consultations.^56^ Adolescent participants also experienced and feared feelings of embarrassment due to dismissive, unhelpful and negative behaviour and treatment by pharmacists and pharmacy staff.^34^, ^35^, ^36^^,^^49^^,^^53^^,^^56^ Asking repetitive or intrusive questions that did not relate to the provision of ECP were perceived by adolescents as lacking empathy and stigmatising, 3% of calls in a mystery caller study across four states in the US found they were asked intrusive questions and between 2% and 6% had to repeat themselves to different staff members.^48^ These concerns were also evident in four mystery client studies that assessed ECP provision.^38^^,^^41^^,^^42^^,^^51^ Pharmacy staff appeared not to find it acceptable to provide ECP to adolescents—they were often dismissive or refused to provide relevant information or supply the ECP.^38^^,^^41^^,^^42^^,^^51^

Adolescents generally believed that the pharmacy setting provided them with greater privacy and confidentiality, as people would not be aware of why they were in the pharmacy, and their parents would be less likely to find out.^32^^,^^41^^,^^49^^,^^55^ In one mystery client study, pharmacists appeared willing to improve access to the ECP by ordering the medicine when it was not in stock.^47^ However, these positive experiences of accessibility were inconsistent. For instance, other studies showed that pharmacy staff indicated to adolescents that confidentiality could not be guaranteed due to various reasons, including the type of medication they were seeking (often ECP) and their age, which created ‘false’ barriers to adolescents’ access.^16^^,^^25^^,^^35^^,^^38^^,^^42^^,^^45^^,^^51^^,^^53^ These differing experiences of privacy and confidentiality were further demonstrated by Wilkinson et al. (2014), where mystery callers posing as 17-year-olds experienced inconsistent responses from pharmacists/pharmacy staff. In some calls, pharmacy staff provided reassurance that the confidentiality of the caller would be maintained due to privacy laws, while others had the impression that their confidentiality could not be maintained when accessing ECP due to their age and the need for parental consent.^41^

Though adolescents felt that pharmacists provided clear and detailed information relevant to medicine use, pharmacists and pharmacy staff were also found to lack correct knowledge around legal restrictions.^32^^,^^39^^,^^46^^,^^52^^,^^56^ In a survey in the US, adolescents were very satisfied with their ECP access experience, with 99% indicating that pharmacists provided clear or very clear information about the ECP.^32^ However, pharmacists and pharmacy staff in eight studies were found to have held incorrect knowledge when it came to age restrictions, the need for parental consent and who can purchase medications.^15^^,^^35^^,^^38^^,^^41^^,^^43^, ^44^, ^45^^,^^47^ In four mystery shopper studies, the manner and level of information and clinical quality provided varied depending on who the mystery shopper was (adolescent female, adolescent male, physician or researcher).^15^^,^^38^^,^^45^^,^^47^

The layout of the pharmacy also influenced how adolescents viewed the accessibility of SRH care in community pharmacies. The layout of the pharmacy did not always provide the level of privacy that adolescents required to maintain their confidentiality when discussing SRH issues.^26^^,^^31^^,^^34^^,^^41^^,^^42^^,^^46^^,^^49^^,^^56^ In one study, an 18-year-old participant highlighted that they are often surrounded by other patients and retail customers when they go to the pharmacy to access their contraception and are, therefore, unable to have a truly private conversation with pharmacists.^49^

Adolescents encountered a lack of uniformity in care across different pharmacies and pharmacists (e.g., pharmacists asking adolescents questions directly versus asking them to complete a form when accessing ECP). The discrepancy between pharmacies or within the same pharmacy in the provision of care created confusion and feelings of uncertainty, preventing them from wanting to return to the pharmacy again.^34^^,^^36^^,^^56^

Adolescents voiced a preference for and experienced feelings of greater ease when engaging with a female pharmacist or pharmacy staff member when accessing contraception and ECP.^26^^,^^49^^,^^56^ Similarly, pharmacy staff in a focus group study also recognised that adolescents may feel more comfortable interacting with female pharmacists.^55^ Female pharmacists were perceived by adolescents to be more understanding of what they were going through, and that adolescent girls would feel more comfortable seeking information and contraception from them.^26^^,^^49^^,^^56^ Although, in one study, male pharmacists were thought to be a neutral source of information^56^; however, adolescents and pharmacists stated that girls may not be comfortable interacting with a male pharmacist, creating a barrier to access.^49^^,^^55^^,^^56^

Empathetic care provided by pharmacists and pharmacy staff made adolescents in three studies feel more comfortable when accessing SRH services. This included approachability of staff, awareness of stigma encountered by adolescents accessing contraception,^46^ promoting a non-judgemental and respectful environment, being mindful of tone and facial expressions^49^ and maintaining adolescents’ discretion.^56^ Mechanisms that pharmacists identified that could address adolescents’ concerns and support a positive experience were providing appointment times, which could remove any discomfort adolescents may feel when requesting a contraceptive consultation.^55^

Pharmacists and pharmacy staff did recognise the importance of treating adolescents with respect and empathy. Through focus groups in the US, pharmacists acknowledged that they need to avoid judgement or making assumptions about the needs of their adolescent patients.^55^ Pharmacists in three survey studies were asked about their level of comfort in dispensing ECP or regular contraceptive methods to adolescents without a doctor’s prescription. In the two ECP-focused studies, only a minority of pharmacists reported feeling discomfort with dispensing ECP for adolescents.^25^^,^^54^ In the one study that focused on regular contraceptive methods, 50% of pharmacists reported discomfort in dispensing contraception for adolescents.^17^ In case scenarios, pharmacists were the most uncomfortable providing ECP to younger adolescents (14-year-olds) and were more likely to seek parental consent.^25^

## Discussion

This systematic review examined the experience and acceptability of contraception and abortion services provided to adolescents in the community pharmacy setting in high-income countries, from both adolescent and pharmacist/pharmacy staff perspectives. The main findings from this review were that adolescents experienced and feared embarrassment, judgement and stigma and had concerns about their confidentiality and privacy. Despite these concerns, adolescents perceived the pharmacy as an accessible and convenient source of SRH services and that pharmacists provided them with comprehensive information. From the pharmacists’ perspective, their attitudes and comfort regarding adolescent sexual activity and contraception provision (including ECP), along with their need for further training, appeared to influence their acceptability to provide these services. While 34 studies met the review inclusion criteria, there were limited studies that focused specifically on the adolescent population. The information in this systematic review pertaining to adolescents was mainly extracted from quotes attributed to adolescent participants, disaggregated quantitative data, or where pharmacy staff were asked specific questions related to providing care and dispensing to adolescents. Adolescents were mainly included as part of studies looking at young people or people of reproductive age. None of the included studies focused on abortion services in the community pharmacy.

The majority of studies included in this review looked specifically at the provision of ECP, and of the seven countries represented in the included studies, the majority were conducted in the US. This may be attributed to legislative changes and variations in state- and federal-level policies regarding ECP provision over-the-counter and pharmacist-prescribing of oral contraceptive pills. Accessible ECP is crucial for preventing unintended pregnancies, reducing the need for abortion, and empowering adolescents to make informed reproductive health decisions. In the US, between 2006 and 2014, multiple legislative reforms took place, transitioning ECP availability from prescription by a medical professional to over-the-counter, along with the removal of age and gender restrictions.^59^^,^^60^ The majority of the studies included in this review focused on provision practices and experience of care,^15^^,^^16^^,^^33^, ^34^, ^35^^,^^37^^,^^38^^,^^41^, ^42^, ^43^^,^^45^^,^^47^^,^^50^, ^51^, ^52^^,^^54^^,^^56^ to identify persistent access barriers and determine the specific obstacles adolescents continue to face. This evidence is critical for advocacy and the development of targeted interventions, particularly concerning adolescent care, that can be integrated into pharmacy practice and policy to improve accessibility. Given ECP’s significance and legislative changes, both nationally and at the state level, may explain the heightened research interest in ECP access in the US and other high-income countries. An increasing number of countries are expanding ECP availability, transitioning from prescription-only dispensing to behind-the-counter and over-the-counter access in pharmacies.^61^^,^^62^ This trend reflects a growing global effort to enhance and better understand ECP access. Variability in findings highlights differences in legislative and practice environments and suggests, particularly in U.S.-based studies, inconsistencies in the delivery of care and awareness of up-to-date policy and practice guidelines.

Previous reviews have looked at the provision of sexual and reproductive services in the pharmacy setting but not specifically at adolescents. The systematic review by Gonsalves and Hindin (2017) looked at young people’s (≤25 years) access, use and quality of care of SRH commodities in pharmacies^20^ while a scoping review by Buckingham et al. (2021) looked at identifying pharmacy-based initiatives that address unintended pregnancy.^63^ Unlike Gonsalves and Hindin, Buckingham et al. and this review only included studies from high-income countries. Although the population and focus of these three reviews differed, there were similarities in findings. The majority of studies that were identified across all three reviews looked at the provision of ECP, with most studies evaluating the provision of services post-legislative change to determine their experience of service delivery and whether there was improvement in access. Unlike the findings from this review and Gonsalves and Hindin, evidence presented by Buckingham et al. showed that the main benefits of pharmacy initiatives from patients’ perspectives were convenience and potential for improved anonymity.^63^ While all three reviews identified convenience as a benefit of the pharmacy setting, there was a lack of consensus in this review and Gonsalves and Hindin around whether anonymity exists in the pharmacy.

Adolescent-friendly care is not about creating a new population-targeted health service but improving the service structure and provider competency to effectively support adolescents’ needs.^64^ Evidence from this review suggests that while community pharmacies can serve as acceptable settings for adolescent contraception services, this is not always the case. Although regulations for ECP and other hormonal contraceptives prescribing are easing across high-income countries, and over-the-counter dispensing of hormonal contraceptives can likely improve access for adolescents, the evidence indicates that adolescents still struggle to access these medications through pharmacies. Furthermore, the care provided within community pharmacies does not consistently align with the Ambresin et al. (2013) adolescent-friendly care framework, which comprises eight domains.^11^ Mapping the evidence from this review against the framework highlights key gaps in service delivery and care. Many of the acceptability and experience findings related to the ‘Staff attitude’ domain. Relevant indicators of ‘staff attitude’ are possessing accurate knowledge, delivering holistic care, demonstrating respect, support, honesty, trustworthiness, and friendliness.^11^ Pharmacists and pharmacy staff frequently failed to meet these indicators, as they were often unsupportive, provided inaccurate information in regards to adolescents’ ability to access to contraception (including ECP), and allowed their personal beliefs to influence whether they provided adolescents with SRH care.^15^^,^^16^^,^^25^^,^^34^^,^^35^^,^^37^^,^^38^^,^^41^^,^^42^^,^^45^^,^^51^^,^^53^ Many adolescents either experienced or feared judgement from pharmacy staff,^31^^,^^34^^,^^36^^,^^49^^,^^56^ highlighting the necessity for enhanced pharmacist education and training to cultivate a more supportive and nonjudgmental environment. This also closely relates to the ‘communication’ domain, which encompasses clarity of information provided, active listening, and the tone of interactions between pharmacists and adolescents.^11^ While evidence from this review indicates that pharmacists were generally regarded as credible sources and provided adolescents with detailed information,^32^^,^^39^^,^^46^^,^^49^^,^^52^^,^^56^ some adolescents reported experiencing intrusive or inappropriate questioning that did not align with their healthcare needs.^38^^,^^41^^,^^42^^,^^48^^,^^51^ Effective communication fosters trust and encourages adolescent engagement with pharmacy services, whereas poorly executed interactions contribute to uncertainty and negative experiences. Additionally, the pharmacy was seen as both conducive (e.g., people go to the pharmacy for a variety of reasons, pharmacist/pharmacy staff do not know you)^32^^,^^41^^,^^49^^,^^55^ and hindering (e.g., out in the open, surrounded by other patients and retail staff)^26^^,^^31^^,^^34^^,^^41^^,^^42^^,^^46^^,^^49^ in maintaining adolescents’ anonymity and confidentiality. These findings, mapped to the ‘age-appropriate environment’ and ‘accessibility of health care’ domains, suggest that while pharmacies offer certain advantages, improvements are needed to fully align with the adolescent-friendly care framework. Furthermore, interpersonal and environmental factors contributed to experiences of embarrassment or concerns about experiencing embarrassment, a key deterrent to accessing services in a pharmacy setting.^31^^,^^34^, ^35^, ^36^^,^^49^^,^^56^ Mixed evidence across studies suggests inconsistency in service delivery, which needs to be addressed to ensure adolescents feel safe when accessing SRH services in pharmacies. Adolescents must also be confident that they will receive the level of respect, privacy/confidentiality and quality of care that they deserve. These findings emphasise the need to strengthen pharmacy environments, enhance pharmacists’ confidence in adolescent care, and improve their ability to provide non-judgmental, informed support—critical steps toward more equitable and effective SRH service provision.

Although pharmacists were viewed by adolescents as knowledgeable,^32^^,^^46^^,^^56^ adolescent-friendly care appears to be an underemphasised or neglected aspect of pharmacist training and service provision. Pharmacists may lack understanding of dispensing regulations for adolescent populations, and they identified the need for further training to improve their comfort and confidence when delivering services to adolescents.^17^^,^^25^^,^^26^^,^^55^^,^^58^ Similarly, a 2017 review of youth-friendly primary care services recognised that health professionals working in primary care (mainly general practitioners) identified the need for additional training to provide services to adolescents and that training of primary care professionals can improve adolescent-friendliness of care.^65^ For some pharmacists, however, it was unclear whether they were denying access due to personal beliefs, a lack of understanding of current guidelines and regulations and/or their discomfort consulting with adolescents. Nonetheless, pharmacists’ and pharmacy staff’s personal biases and/or general discomfort in providing care to adolescents appear to contribute to uncomfortable experiences for adolescents or refusal of service.^37^^,^^38^^,^^40^, ^41^, ^42^^,^^51^

The availability of further training (on both technical and interpersonal aspects of adolescent-friendly care) and ongoing implementation support would provide pharmacists with the knowledge and communication skills to appropriately and empathetically respond to the needs of adolescents. For instance, the ALLIANCE trial^66^ (a randomised controlled trial currently underway in community pharmacies in two Australian states) employs a model that expands community pharmacists’ scope of practice to deliver contraceptive counselling and refer patients to prescribers of contraception (e.g., general practitioner, nurse practitioner or sexual health clinic). The ALLIANCE trial community pharmacists are upskilled to provide person-centred contraceptive counselling through a suite of previously tested upskilling activities,^67^ which include an online educational module, educational outreach through academic detailing, and support through a virtual community of practice. Based on the findings of this review and other relevant literature,^68^ incorporating adolescent-focused activities as part of these upskilling strategies^68^ is necessary to ensure that this and similar interventions improve access for adolescents. To ensure consistency of service, all pharmacists need to be up to date with and follow the current guidelines to minimise arbitrary barriers to access at the point of care.

A key strength of this study is that both community pharmacists’ and adolescent perspectives were examined, highlighting both barriers to access and provision that are specific to adolescent patients. Previous reviews focused on the broader population and outcomes—this is the first review that looks specifically at contraceptive and abortion care for adolescents aged 10–19 years. A limitation of this review was that few included studies focused specifically on the adolescent population, although several mystery client studies that simulated patients were ‘adolescents’ or a ‘health professional calling on behalf of an adolescent patient’. There were limited data on the experience of younger adolescents, particularly those under 16 years. This review was limited to studies published in English, which may have resulted in the exclusion of relevant research available in other languages. Additionally, given that the included studies span a period of 25 years, changes in service delivery models and evolving perceptions over time may influence the applicability of the findings to contemporary healthcare contexts. Finally, findings of this review cannot be generalised to abortion care, as no studies regarding abortion care were identified, but they may provide some insight into the challenges young people may face when attempting to access abortion services from pharmacies.

This review identified that community pharmacies can be safe and accessible settings where adolescents can conveniently seek clinical and dispensing services for contraception. However, no studies regarding adolescent-centred abortion care in community pharmacies were identified. The findings show that both the pharmacy setting and pharmacists’ competency in adolescent-friendly care need to be optimised to further improve consistency of service and, in turn, improve access and appropriateness for adolescents. Therefore, it is necessary to develop and evaluate adolescent-centred SRH training and implementation support for pharmacists to ensure high-quality SRH are provided to adolescents seeking SRH care.

## Contributors

AA is responsible for the conceptualization of the study, screening all identified abstracts, reviewed all identified articles, extracted the data from articles, and assessed the risk of bias. AA and DM contributed to the data interpretation and review, and editing. AA drafted the initial manuscript. DM significantly edited and critically reviewed the manuscript. All authors approved the final version of the manuscript.

## Data sharing statement

This manuscript includes a tertiary use of data. Primary data sources are already published and openly available.

## Declaration of interests

AA reports no conflict of interest. DM has received research funding and has been an advisory board member for Bayer and Organon (manufacturers of LARC).
