# Supplementary material for: Contraception and abortion information and care in community pharmacy for adolescents: a systematic review
Source: eClinicalMedicine. 2025 Jul 31;86:103394. doi: 10.1016/j.eclinm.2025.103394 (PMC12336644; doi:10.1016/j.eclinm.2025.103394)
Supplement: Supplementary Table S1 [file mmc1.docx]

**Supplementary Material**

**Article title:** Contraception and abortion information and care in community pharmacy for adolescents: a systematic review

**Supplementary Table 1.** JBI Critical Appraisal of included articles

**Supplementary table 1.** JBI Critical Appraisal of included articles

| **Qualitative studies** | **Congruity between the stated philosophical perspective and the research methodology** | **Congruity between the research methodology and the research question or objectives** | **Congruity between the research methodology and the methods used to collect data** | **Congruity between the research methodology and the representation and analysis of data** | **Congruity between the research methodology and the interpretation of results** | **Statement locating the researcher culturally or theoretically** | **Influence of the researcher on the research. and vice- versa. addressed** | **Participants. and their voices. adequately represented** | **Research ethical according to current criteria or. for recent studies, and is there evidence of ethical approval by an appropriate body** | **Conclusions drawn in the research report flow from the analysis. or interpretation. of the data** | **Score** |
| --- | --- | --- | --- | --- | --- | --- | --- | --- | --- | --- | --- |
| Barrense-Dias et al. 2022 | Yes | Yes | Yes | Yes | Yes | No | No | Yes | Yes | Yes | 80 |
| Glasier et al. 2021 | Yes | Yes | Yes | Yes | Yes | Yes | No | Yes | Yes | Yes | 90 |
| Hobbs et al. 2009 | Yes | Yes | Yes | Yes | Yes | Yes | No | Yes | Yes | Yes | 90 |
| Meredith et al 2020# | Yes | Yes | Yes | Yes | Yes | Yes | No | Yes | Yes | Yes | 90 |
| Sampson et al. 2009# | Yes | Yes | Yes | Yes | Yes | Yes | Yes | Yes | Yes | Yes | 100 |
| Wilkinson et al. 2014 | Yes | Yes | Yes | Yes | Yes | No | No | Yes | No | Yes | 70 |
| Wilkinson, Miller et al. 2018 | Yes | Yes | Yes | Yes | Yes | Yes | No | Yes | Yes | Yes | 90 |
| Wollum et al. 2020# | Yes | Yes | Yes | Yes | Yes | No | No | Yes | Yes | Yes | 80 |
| Zuniga et al. 2019 | Yes | Yes | Yes | Yes | Yes | Yes | No | Yes | Yes | Yes | 90 |
| **Cross-sectional studies** | **Criteria for inclusion in the sample clearly defined** | **Study subjects and the setting described in detail** | **Exposure measured in a valid and reliable way** | **Objective, standard criteria used for measurement of the condition** | **Confounding factors identified** | **Strategies to deal with confounding factors stated** | **Outcomes measured in a valid and reliable way** | **Appropriate statistical analysis used** |  |  |  |
| Ashcraft et al. 2020 | Yes | Yes | Yes | Yes | Unclear | Yes | Yes | Yes |  |  | 88 |
| Ashcraft et al. 2022 | Yes | Yes | Yes | Yes | Unclear | Yes | Yes | Yes |  |  | 88 |
| Ashcraft et al. 2023 | Yes | Yes | Yes | Yes | Unclear | Yes | Yes | Yes |  |  | 88 |
| Cleland et al. 2016 | No | Yes | Yes | Yes | No | No | Yes | Yes |  |  | 75 |
| Conard et al. 2003 | Yes | Yes | Yes | Yes | Unclear | Yes | Yes | Yes |  |  | 88 |
| Gomez et al. 2022 | Yes | Yes | Yes | Yes | Yes | Yes | Yes | Yes |  |  | 100 |
| Grindlay et al 2023 | Yes | Yes | Yes | Yes | Unclear | Yes | Yes | Yes |  |  | 88 |
| Horsfield et al. 2014 * | No | Yes | Yes | Yes | No | No | Yes | Yes |  |  | 63 |
| Hsu et al 2020 | Yes | Yes | Yes | Yes | Unclear | Yes | Yes | Yes |  |  | 88 |
| Hussainy et al. 2015 | Yes | Yes | Yes | Yes | No | No | Yes | Yes |  |  | 75 |
| Khorsandi et al. 2021 | Yes | Yes | Yes | Yes | Unclear | No | Yes | Yes |  |  | 75 |
| Lewington & Marshall 2006 | Yes | Yes | Yes | Yes | Unclear | Yes | Yes | Yes |  |  | 88 |
| Manski & Kottke 2015 | Yes | Yes | Yes | Yes | Yes | Yes | Yes | Yes |  |  | 100 |
| Meredith et al. 2020# | No | Yes | Yes | Yes | Yes | Yes | Yes | Yes |  |  | 88 |
| Parsons et al. 2013 | Yes | Yes | Yes | Yes | Unclear | No | Yes | Yes |  |  | 75 |
| Richman et al. 2012 | Yes | Yes | Yes | Yes | No | No | Yes | Yes |  |  | 75 |
| Ritter et al. 2018 | Yes | Yes | Yes | Yes | Yes | Yes | Yes | Yes |  |  | 100 |
| Rubin et al. 2011 | Yes | Yes | Yes | Yes | Yes | No | Yes | Yes |  |  | 88 |
| Sampson et al 2009# | No | Yes | Yes | Yes | Yes | Yes | Yes | Yes |  |  | 88 |
| Soper & Di Meglio 2020 | Yes | Yes | Yes | Yes | Unclear | No | Yes | Yes |  |  | 75 |
| Stone et al. 2020* | No | Yes | Unclear | Yes | Unclear | Yes | Unclear | Yes |  |  | 50 |
| Sucato et al. 2001 | Yes | Yes | Yes | Yes | Yes | Yes | Unclear | Unclear |  |  | 75 |
| Uysal et al. 2019 | Yes | Yes | Yes | Yes | Yes | Yes | Yes | Yes |  |  | 100 |
| Wilkinson et al, 2012 | Yes | Yes | Yes | Yes | Yes | Yes | Yes | Yes |  |  | 100 |
| Wilkinson et al. 2017 | Yes | Yes | Yes | Yes | Yes | Yes | Yes | Yes |  |  | 100 |
| Wilkinson, Rafie et al. 2018 | Yes | Yes | Yes | Yes | Yes | Yes | Yes | Yes |  |  | 100 |
| Wilson & Williams 2000 | Yes | Yes | Yes | Yes | Yes | Yes | Yes | Yes |  |  | 100 |
| Wollum et al. 2020# | Yes | Yes | Yes | Yes | No | No | Yes | Yes |  |  | 75 |

# Mixed-method study, the qualitative and quantitative aspects of the study were assessed using the appropriate JBI tools

*Moderate methodological quality. All other studies were assessed as having high methodological quality. Low quality studies are determined if 0-49% of responses are “yes”, moderate quality if 50-69% of responses are “yes” and high quality if 70-100% responses are “yes”.
